# The Effect of *α*‐Mangostin on the Pharmacokinetic Profile of Tofacitinib in Rats Both In Vitro and In Vivo

**DOI:** 10.1002/bdd.70012

**Published:** 2025-08-16

**Authors:** Jiange Yao, Zebei Lu, Quan Zhou, Abdullah Al Mamun, Yaru Shi, Shuanghu Wang, Ying Yao

**Affiliations:** ^1^ Department of Outpatient The Fifth Affiliated Hospital of Wenzhou Medical University Lishui China; ^2^ Key Laboratory of Joint Diagnosis and Treatment of Chronic Liver Disease and Liver Cancer of Lishui Central Laboratory of The Lishui Hospital of Wenzhou Medical University The First Affiliated Hospital of Lishui University Lishui People's Hospital Lishui China

**Keywords:** *α*‐mangostin, herb–drug interactions, pharmacokinetics, tofacitinib

## Abstract

This study investigated the effects of *α*‐mangostin (*α*‐MG) on the pharmacokinetics of tofacitinib in vitro and in vivo, aiming to recommend its appropriate application in clinical practice. To investigate the values of IC_50_ and inhibition of *α*‐MG in vitro, rat liver microsomes were incubated with tofacitinib. In this study, Sprague–Dawley rats were randomly assigned to three groups: a control group, a single‐dose group (50 mg/kg of *α*‐MG), and a multiple‐dose group (50 mg/(kg/d) of *α*‐MG for 7 days). Tofacitinib (10 mg/kg) was administered 30 min after the intervention of *α*‐MG to each group. The plasma was collected from the caudal vein at different time points and in heparinized tubes. Tofacitinib metabolites in the plasma were determined by UPLC‐MS/MS. Further analyses were conducted utilizing Pymol molecular docking simulation to evaluate the effect of *α*‐MG on tofacitinib. Our results showed that MG inhibited the metabolism of tofacitinib in vitro by exhibiting both competitive and noncompetitive inhibition. More importantly, we found that multiple‐dose administration of *α*‐MG significantly increased the AUC_(0–12h)_, AUC_(0–∞),_ and C_max_, prolonged the *t*_1/2_ and shortened the MRT_(0–12h)_ and MRT_(0–∞)_ of tofacitinib. At the same time, the CL_z/F_ was decreased, which was consistent with the results of in vitro experiments. Furthermore, we observed no significant difference between single‐dose and multiple‐dose groups. Intriguingly, *α*‐MG and tofacitinib were close at the CYP3A4 spatial location. In summary, our investigation demonstrated that *α*‐MG significantly impacts the metabolism of tofacitinib both in vitro and in vivo, suggesting potential herb–drug interactions (HDIs). The use of tofacitinib with herbs containing MG should be monitored clinically.

## Graphical abstract

*α*‐mangostin (*α*‐MG) significantly affected tofacitinib metabolism by competing for the same site in CYP3A4. Co‐administration of these two medicines poses a potential herb–drug interaction.

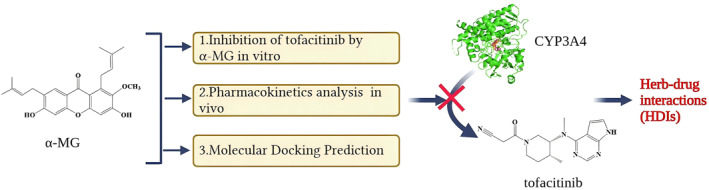

## 1 Introduction

Tofacitinib, a newly developed, orally available small molecule inhibitor of the Janus kinase (JAK) family, predominantly, and selectively inhibits JAK1 and JAK3 and binds to JAK2 and Tyk2 (Harrington et al. 2020). Tofacitinib has been approved for the treatment of moderate‐to‐severe rheumatoid arthritis (RA) resulting from intolerance to methotrexate (Singh et al. 2016). Tofacitinib belongs to the class of drugs that alleviate the pathogenesis and progression of a variety of diseases. Accumulating evidence indicates that tofacitinib ameliorates the dysregulation of the immune system and alleviates inflammatory response by obstructing the JAK signaling pathway and attenuating the activation of JAK‐mediated cytokines including IL‐2, ‐4, ‐7, ‐9, ‐15, and ‐21 (Dhillon 2017) (F. Wang et al. 2020). However, adverse reactions, particularly infections are commonly associated with its use (Winthrop 2017). Rheumatoid arthritis (RA) is an autoimmune disease characterized by joint deformities, chronic inflammation and progressive impairment. Tofacitinib is widely used despite the widespread use of RA therapies due to the reduced risks of allergies, infections, and other adverse effects (Khojah et al. 2018; Chandran and Goel 2012; Wu et al. 2022).

Tofacitinib is rapidly absorbed with 74% absolute bioavailability. Approximately 70% of the dose was metabolized by the liver and 30% was excreted in the urine after renal filtration and maternal drug secretion (Dowty et al. 2014). Tofacitinib metabolism involves CYP3A4 primarily with a small amount modified by 2C19, 2D6, and 1A2 (Vyas et al. 2013; Guo et al. 2019). The pharmacokinetics of tofacitinib are affected by CYP450‐mediated drug interactions, resulting in individual differences in plasma concentrations. Rifampicin and fluconazole are moderately effective CYP3A4 inhibitors that increase tofacitinib exposure. Previous studies indicated that these variances required dosage adjustments for tofacitinib (Veeravalli et al. 2020).

The natural xanthone *α*‐mangostin (*α*‐MG) from mangosteen (*Garcinia mangostana* Linn) pericarp has been used to alleviate abdominal pain, diarrhea, chronic ulcers, and infected wounds (Herrera‐Aco et al. 2019). Numerous research investigations have reported that MG possesses a variety of bioactive compounds including anti‐oxidants, anti‐inflammatory agents, anti‐bacterial agents, anti‐virals, and antitumor agents (Chen et al. 2018). In addition, *α*‐MG has been identified as a promising drug candidate for the treatment and management of RA. Several studies have also shown that *α*‐MG inhibits RA progression by enhancing ROS accumulation, alleviating RA fibroblast‐like synoviocyte apoptosis and mitigating inflammatory cytokine production (J. Zuo et al. 2018; Zhang et al. 2022; Sheng et al. 2019).

CYP450 enzymes metabolize herbs or plant extracts, resulting in herb–drug interactions (HDIs) (H. Zuo et al. 2022). It has been demonstrated that tofacitinib induces HDIs with quercetin, resveratrol, and Shaoyao–Gancao–Fuzidecoction (B. Wang et al. 2020; L. Lin et al. 2022; Ye et al. 2023). Investigating the interaction between tofacitinib and *α*‐MG is crucial due to their potential effects in treating RA.

This study examined the pharmacokinetics of tofacitinib in rat liver microsomes (RLM) and Sprague–Dawley rats (SD). The pharmacokinetic properties of tofacitinib in vitro and in vivo for both multiple‐dose and single‐dose pretreatment with *α*‐MG were analyzed using a sensitive and reliable UPLC/MS‐MS system. In addition, the potential mechanism between *α*‐MG and tofacitinib interaction was identified by Pymol molecular docking simulation. Our data demonstrated the existence of HDIs between *α*‐MG and tofacitinib. Thus, this study provides crucial information on the safety of *α*‐MG with tofacitinib for clinic application.

## 2 Materials and Methods

### 2.1 Chemicals and Reagents

Tofacitinib (purity > 98%) and the metabolite of tofacitinb (M8) were purchased from Beijing Sunflower and Technology Co. Ltd. (Beijing, China). *α*‐MG (purity > 98%) was purchased from Chengdu Manster Biotechnology Co. (Chengdu, China). Acetonitrile and methanol were of liquid chromatography grade and were obtained from Fisher Scientific Co. (Fair Lawn, NJ). Ultra‐pure water was produced with the Milli–Q system in the laboratory (Millipore, Bedford, MA, USA). All other chemicals and reagents were of analytical quality or higher.

### 2.2 Animals and Treatment

Healthy SPF‐grade SD male rats (average weight 250 ± 20 g) were supplied by the experimental animal center of Wenzhou Medical University (Wenzhou, China). The rats were randomly allocated into three groups of five each and standardly maintained at 20°C–25°C, 60% ± 5% humidity with a 12 h/12 h dark–night cycle. All rats were allowed free access to water and diet without restrictions and acclimatized feeding for 2 weeks before initiating the animal experiments. The experimental protocols were reviewed and approved by the Experimental Animal Ethics Committee of Wenzhou Medical University, Zhejiang, China (Approval No. wydw 2019‐650).

### 2.3 Instrument and Conditions

Plasma samples were analyzed by ultra‐performance liquid chromatography–tandem mass spectrometry (UPLC‐MS/MS) utilizing a Waters XEVO TQD triple quadrupole mass spectrometer and electrospray ionization source. The chromatographic separation was performed on the ACQUITY UPLC HSS T3 column (100 mm × 2.1 mm, 1.8 μm) maintained at 40°C. The flow rate was 0.4 mL/min and the injection volume was 5 μL. The mobile phase comprises acetonitrile (A) and pure water (containing 0.1% formic acid, B). The gradient elution was set as follows: 0–0.5 min, a linear gradient of 10%–30% A; 0.5–1 min, a linear gradient to 95% A; 1–2 min, an isocratic gradient at 95% A; 2–2.3 min, decreased linearly to 10% A. The total time required for analysis was 3 min.

Analytes were detected using multiple reaction monitoring in positive ion mode. The precursor ion and product ion were m/z 313.18 → 149.03 for tofacitinib, m/z 299.162 → 98.0969 for tofacitinib M8, and m/z 325.98 → 291.07 for midazolam (internal standard, IS). We determined the optimal MS parameters: cone voltage (40 V for tofacitinib, 40 V for tofacitinib M8, and 50 V for midazolam) and collision energies (30 V, 30 and 26 V). Data collection and instrument control were performed using MassLynx software (Version 4.1, Waters Co., MA).

M8, a characteristic metabolite generated during tofacitinib metabolism in both humans and rats, is most abundant in rats (Dowty et al. 2014; Liu et al. 2021). Therefore, M8 serves as an appropriate candidate for investigating tofacitinib pharmacokinetics.

### 2.4 Metabolic Inhibition of *α*‐MG on Tofacitinib In Vitro

RLMs were prepared by utilizing differential centrifugation as previously described (Hong et al. 2022). Tofacitinib was incubated in a 200 μL system with 40 μM (K_m_ value), 0.5 mg/mL RLM, 100 mM PBS buffer (pH = 7.4), and various concentrations of *α*‐MG (final concentration). The above procedures were performed on ice in three parallel groups. After vortexing and mixing, the reaction was pre‐incubated at 37°C for 5 min. The reaction was next initiated by adding 10 mL of NADPH (1 mM) at 37°C for 60 min and all the reactions were terminated at −80°C. Finally, 200 μL of acetonitrile (containing 200 ng/mL of midazolam) was added. To detect and quantify tofacitinib metabolite M8, the supernatant was gently mixed for 30 s and centrifuged at 13,000 rpm for 5 min. The value of IC_50_ was finally calculated using GraphPad Prism software (Version 9.0, GraphPad Software Inc., San Diego, CA).

To investigate the mechanisms of *α*‐MG inhibition by tofacitinib, *α*‐MG and tofacitinib were incubated in a 200 μL system with Km and IC_50_ values of 10, 20, 40, and 50 μM, respectively. The incubation procedure used in this study was identical to the one described before.

### 2.5 Pharmacokinetic Interactions Between *α*‐MG and Tofacitinib In Vivo

To evaluate the effects of *α*‐MG on the pharmacokinetics of tofacitinib, 15 rats were randomly assigned to three groups: control, single‐dose (50 mg/kg *α*‐MG), and multiple‐dose (50 mg/kg/d *α*‐MG for 7 days). The control group was given normal saline for 7 days; the single‐dose group was given saline for 6 consecutive days, followed by administration of *α*‐MG 50 mg/kg on the 7th day. The multi‐dose group was administered 50 mg/kg of *α*‐MG for 7 successive days. The rats were pretreated with the above pretreatment for 30 min before receiving 10 mg/kg of tofacitinib on day 7. Then, the blood from the tail vein was collected from each group at 0.083, 0.5, 1, 2, 3, 4, 6, 8, 10, and 12 h. The plasma samples were collected and mixed with 200 acetonitrile‐containing IS during the previous step. The mixture was then vortexed for 10 s and centrifuged at 13,000 rpm for 10 min. The supernatant was finally analyzed by mass spectrometry with a volume of 5 μL. The doses of *α*‐MG and tofacitinib (50 mg/kg and 10 mg/kg, respectively) administered to the rats were selected based on published data. The 50 mg/kg *α*‐MG dose was chosen for its efficacy in treating rat arthritis and achieving adequate blood and tissue concentrations (Xu et al. 2017; Hu et al. 2021). Moreover, the pharmacokinetic comparisons indicate that the first‐pass metabolism ratio of tofacitinib at 10 mg/kg in rats closely resembles that observed in humans, justifying the selection of this dose for the current study (Lee and Kim 2019).

### 2.6 Molecular Docking Method

The crystal structure of CYP3A4 was retrieved from the RCSB PDB database (https://www.rcsb.org/) and the molecular structures of tofacitinib and *α*‐MG were retrieved from PubChem (https://pubchem.ncbi.nlm.nih.gov/), respectively. We performed molecular docking and structure selection utilizing Pymol (Version 2.5.2) and AutoDock Vina (Version 1.2.0). The results from the docking visualization revealed the hydrogen bonding location for *α*‐MG interaction with CYP3A4.

### 2.7 Statistical Analysis

GraphPad Prism software (Version 9.0, GraphPad Software Inc., San Diego, CA) was utilized to determine the kinetic parameters of tofacitinib including Lineweaver–Burk plots, plasm concentration–time curves and IC_50_ values. The main pharmacokinetics parameters such as half‐life (*t*_1/2_), peak concentration (C_max_), area under the drug–time curve (AUC), clearance (C_L/F_), the mean residence time (MRT), and the apparent volume of distribution (V_z/F_) were calculated by fitting with Drug and Statistics (DAS) software (Version 3.2.8, The People's Hospital of Lishui, Zhejiang Province, China). All data were expressed as the mean ± standard deviation. Statistical analysis was performed on the independent sample utilizing SPSS software (Version 22.0, SPSS Inc., USA). *p* < 0.05 was considered to be statistically significant.

## 3 Results

### 3.1 Effects of *α*‐MG on the Pharmacokinetics of Tofacitinib In Vitro

**FIGURE 1:**
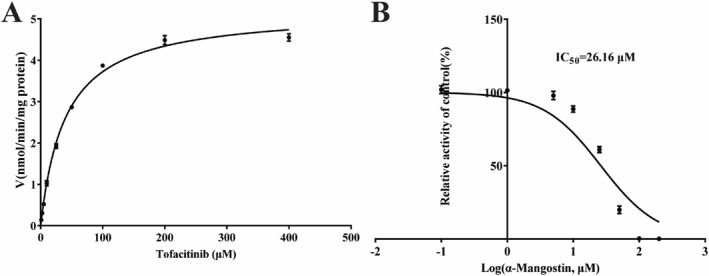
Michaelis–Menten kinetics (A) and the IC_50_ value (B) of tofacitinib in RLMs.

**FIGURE 2:**
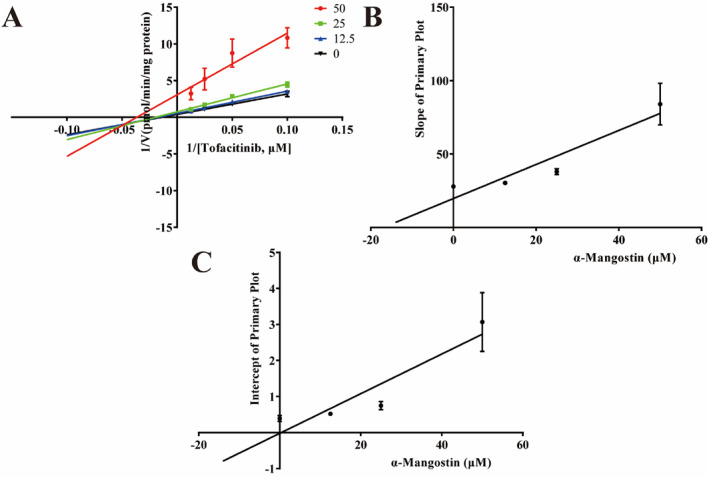
Lineweaver–Burk plots for *α*‐MG inhibition of tofacitinib metabolism in RLMs. (A) Lineweaver–Burk plot for *α*‐MG inhibition of tofacitinib with different concentrations in RLMs. Data are shown with the mean ± SD of three parallel experiments: (B) Slope of primary plot. (C) Intercept of primary plot.

The liver microsomal incubation system was used to investigate the inhibitory mechanism of *α*‐MG on tofacitinib in vitro. Figure 1 shows the V_max_ of 5.210 ng/min/mg, K_m_ of 39.96 μM and IC_50_ of 26.16 μM for tofacitinib. We further conducted enzyme inhibition kinetic analyses to investigate the inhibitory effect of *α*‐MG on tofacitinib. Lineweaver–Burk plot shows a mixed inhibitory impact of *α*‐MG on tofacitinib metabolism with inverses of enzyme reaction rate and substrate concentration intersecting on the negative *X*‐axis. Moreover, Figure 2 of the Lineweaver–Burk secondary plots shows mixed competitive and noncompetitive inhibition at 16.97 and 0.398 μM (*α* = 42.6 ≠ 1).

### 3.2 Effects of *α*‐MG on the Pharmacokinetics of Tofacitinib In Vivo

**FIGURE 3:**
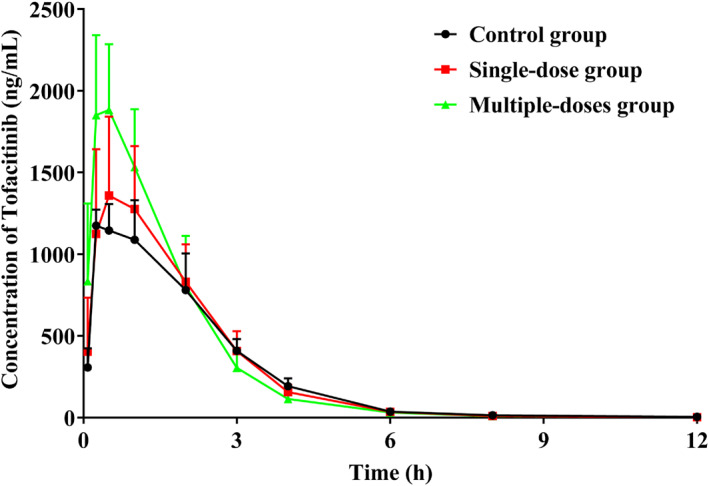
Mean plasma concentration‐time curves of tofacitinib in control, single‐dose, and multiple‐dose groups.

Figure 3 displays the plasma concentration–time curve of tofacitinib in rats pre‐treated with *α*‐MG. In addition, Table 1 presents the detailed pharmacokinetic parameters of the drug. The plasma concentration of tofacitinib reached a maximum of about 0.5 h after oral administration. A single dosage of *α*‐MG pretreatment led to a substantial increase in Cmax and a prolonged *t*_1/2_ of tofacitinib (*p* < 0.05). The results exhibited that multiple‐dose pretreatment with *α*‐MG increased AUC_(0–*t*)_, AUC_(0–∞),_ and C_max_, shorter MRT_(0–*t*)_ and longer *t*_1/2_ of tofacitinib while decreasing CL_z/F_. However, pretreatment with *α*‐MG did not significantly affect the values of *T*_max_ and V_z/F_ of tofacitinib.

**TABLE 1:** Main pharmacokinetic parameters of tofacitinib in vivo (*n* = 5).

Pharmacokinetic parameters	Control group	Single‐dose group	Multiple‐doses group
AUC_(0–*t*)_ (μg/L*h)	3083.565 ± 301.285	3658.646 ± 564.322	4141.095 ± 741.28^a^
AUC_(0–∞)_ (μg/L*h)	3085.624 ± 301.768	3663.569 ± 566.516	4152.785 ± 748.688^a^
MRT_(0–*t*)_ (h)	1.895 ± 0.254	1.71 ± 0.141	1.561 ± 0.132^a^
MRT_(0–∞)_ (h)	1.902 ± 0.258	1.725 ± 0.143	1.596 ± 0.13^a^
*t*_1/2_ (h)	1.064 ± 0.162	1.409 ± 0.217^b^	1.805 ± 0.425^a^
*T*_max_ (h)	0.5 ± 0.306	0.7 ± 0.274	0.45 ± 0.112
V_z/F_ (L/kg)	5.01 ± 0.866	5.601 ± 0.825	6.28 ± 1.054
CL_z/F_ (L/h/kg)	3.269 ± 0.313	2.789 ± 0.48	2.464 ± 0.392^a^
C_max_ (μg/L)	1281.328 ± 96.824	1607.46 ± 208.728^b^	2133.934 ± 508.608^a^

*Note:* All data were assessed for distribution using the Q‐Q plots method and normally distributed data were expressed as mean ± SD.

Abbreviations: AUC, the area under concentration‐time curve; CL_z/F_, the clearance; C_max_, the peak plasma concentration; MRT, the mean residence time; *t*_1/2,_ the half‐life period; *T*_max_, the maximum plasma, maximum blood time; V_z/F_, the apparent volume of distribution.

^a^ *p* < 0.05 indicating a statistically significant difference between the multiple‐dose group and control group.

^b^ *p* < 0.05 indicates a statistically significant difference between the single‐dose group and control group.

### 3.3 Molecular Docking Prediction of *α*‐MG With Tofacitinib

**FIGURE 4:**
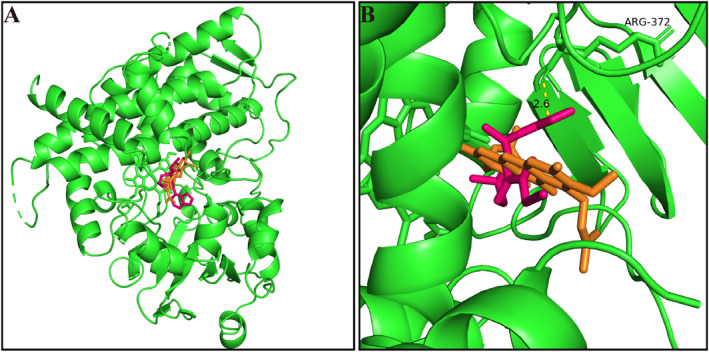
Molecular docking scheme of *α*‐MG and tofacitinib. (A) Docking simulation of *α*‐MG, tofacitinib, and CYP3A4. (B) Action sites and spatial location of *α*‐MG, tofacitinib, and CYP3A4 via hydrogen bonding, where orange represents *α*‐MG and red is tofacitinib.

To comprehend the mechanism of *α*‐MG interaction with tofacitinib, molecular docking analysis was conducted utilizing the previous technique. Pymol simulations found that tofacitinib bonded with CYP3A4 site arginine (ARG) 372 via hydrogen bonding, resulting in 2.6 A of action site, whereas *α*‐MG partially overlapped with tofacitinib in spatial structure (Figure 4). Intriguingly, we found that two drugs are likely to compete due to their proximity in spatial location.

## 4 Discussion

RA is a systemic chronic inflammatory disease that affects every joint in the body, causing cartilage breakdown, bone fractures, and metabolic disorders that reduce the quality of life (Radu and Bungau 2021; Y. J. Lin et al. 2020). Tofacitinib is characterized by excellent pharmacokinetics with limited interactions with other drugs in clinical practice (Veeravalli et al. 2020). Herbs or herbal extracts with CYP activity may cause interactions that increase the risk of HDIs. Studies have indicated that HDIs may be related to the CYP450 enzymes in drug metabolism (Zhao et al. 2021).

MGs exert a variety of pharmacological actions including anti‐inflammatory, antitumor (pro‐apoptotic, anti‐proliferative, anti‐invasive, metastatic, etc.), anti‐lipoatrophy, anti‐oxidation, etc. In addition, *α*‐MG possesses remarkable anti‐inflammatory functions by inhibiting the formation of NO through the suppression of iNOS and several inflammatory factors including IL‐1*β*, TNF‐*α*, NF‐κB, COX‐1, and COX‐2. Accumulating evidence indicates that *α*‐MG regulates various inflammatory conditions by reducing anti‐collagen II IgG2a antibody production, boosting IL‐10 formation, mitigating ROS‐mediated oxidative stress and posing therapeutic potential in combination with anti‐RA drugs (Herrera‐Aco et al. 2019; Chavan and Muth 2021). Previous research suggests that *α*‐MG promotes fibroblast‐like synoviocyte death in RA via the ROS/ERK1/2 pathway, reversing RA development in a dose‐dependent manner (Sheng et al. 2019). In addition, *α*‐MG improves glucose metabolism, inhibits aerobic glycolysis, alleviates hypoxia and ROS, and impairs angiogenesis via the HIF‐1*α*/VEGF signaling pathway (Jiang et al. 2021). The ADMET predictor suggests that *α*‐MG is metabolized by CYP1A2, 2C9, 2C6, and 3A4 enzymes and inhibits CYP2C enzyme and promotes phase‐I metabolism through trioxidation (Rukthong et al. 2020; Foti et al. 2009). Therefore, the therapeutic regimen should be adjusted based on its interaction with the combinations of the tofacitinib drug.

In an in vitro experiment evaluating the effect of u03b1‐MG on tofacitinib pharmacokinetics, Michael's equation was applied to analyze their interaction, yielding the following values: V_max_ (5.210 ng/min/mg), K_m_ (39.96 μM) were similar to the previous reports (Shi et al. 2024). The high IC_50_ value (26.16 μM) obtained after *α*‐MG treatment indicated a low inhibitory potency. In addition, *α*‐MG exerted competitive and noncompetitive inhibition in vitro. According to the in vitro study, the Ki value of *α*‐MG was 16.97 μM, translating to a blood concentration of 6965.5 ng/mL, implying that tofacitinib can only be effectively inhibited at higher concentrations. Previous studies have revealed that MG‐loaded self‐microemulsion significantly improved oral availability with an increase in C_max_ from 734.1 ng/mL to 8066.9 ng/mL at a dose of 50 mg/kg (Xu et al. 2017). Thus, the drug concentration of *α*‐MG at inflammatory therapeutic doses could exert an inhibitory effect. *α*‐MG is abundant in dried mangosteen pericarp, with extraction efficiency demonstrating significant solvent dependence: acetone/water (80:20, v/v) yields 5.5 mg/g *α*‐MG versus 0.36 mg/g via water extraction, where a pure water environment may be more descriptive of its presence in the body (Walker 2007; Bae et al. 2021). Kondo et al. investigated the effect of *α*‐MG on the body of healthy volunteers receiving 59 mL of a xanthone‐rich mangosteen liquid supplement for 1 h (the amount of *α*‐MG was unknown), the C_max_ of *α*‐MG was 3.12 ng/mL, which is much lower than the inhibitory concentration in vivo (Kondo et al. 2009). The daily consumption of *α*‐MG from dried mangosteen pericarp or commercial products is relatively low. However, formulation optimization through tofacitinib administration with or over anti‐inflammatory therapeutic regimens may result in clinically significant pharmacokinetic interactions. There is a lack of clinical data on *α*‐MG and the complex human environment is influenced by a variety of factors such as protein binding and metabolites. Further in vivo pharmacokinetic and pharmacodynamic characterization is required to determine the effect of *α*‐MG on tofacitinib.

This study investigated the effects of *α*‐MG on the pharmacokinetics of tofacitinib in rats. The maximum concentration of tofacitinib in blood was reached within 0.5 h of oral administration. In comparison, when rats were pretreated with *α*‐MG, the *t*_1/2_ of tofacitinib was prolonged from 1.064 ± 0.162 h to 1.409 ± 0.217 h (single‐dose group) and 1.805 ± 0.425 h (multiple‐dose group). The C_max_ was increased from 1607 ± 208.728 μg/L (single‐dose group) to 2133.934 ± 507.608 μg/L (multiple‐dose group). It is worth noting that tofacitinib showed a significant increase in AUC, a 25% decrease in CL_z/F_, and a reduction in MRT in the *α*‐MG multiple‐dose pretreatment group. These data suggested a change in the pharmacokinetics of tofacitinib when coexisting with *α*‐MG and increased systemic exposure at multiple doses.

According to some references, MG inhibits P‐glycoprotein (P‐gp), which may affect tofacitinib the pharmacokinetics (Bae et al. 2021) (Dechwongya et al. 2020). *α*‐MG may depress P‐gp activity by interfering with the activity of the P‐gp ATPase or by downregulating the expression of MDR1, which may lead to a decrease in P‐gp transport proteins, thereby reducing drug efflux and enhancing the bioavailability of P‐gp substrates. Tofacitinib is a substrate of P‐gp. P‐gp is expressed in a variety of organs and *α*‐MG can be targeted to tissues such as the small intestine, liver, and spleen (Xu et al. 2017; Choi 2020). Consequently, the absorption and distribution of tofacitinib may also be differentially influenced by *α*‐MG‐mediated P‐gp modulation. Elevated *t*_1/2_, AUC and C_max_ values indicate increased absorption of tofacitinib. The potentiation of drugs may improve therapeutic efficacy and increase the risks of adverse effects. Previous studies have shown that MRT is generally prolonged due to the prolonged t1/2 (B. Wang et al. 2020; Shen et al. 2020). Interestingly, the multi‐dose group showed a reduction in MRT. In addition, the water extract of mangosteen pericarp enhanced brain delivery of donepezil via inhibition of the P‐gp pathway (Bae et al. 2021). *α*‐MG may accelerate tofacitinib distribution to peripheral tissues, resulting in a reduction in MRT as plasma concentrations rapidly decline. However, comprehensive validation through multicompartment model and tissue distribution studies under tofacitinib‐*α*‐MG co‐administration could be considered to elucidate these mechanistic interactions and optimize clinical translation.

The study also analyzed the binding locations of *α*‐MG and tofacitinib to CYP3A4. About 50% of drugs are metabolized by the liver enzyme CYP3A4 (Guo et al. 2019). In most metabolism‐based HDIs, CYP450 enzymes interact with other drugs for the same enzyme binding site due to xenobiotic substrates. In docking simulations, the CYP3A4 site was bound by tofacitinib, whereas *α*‐MG was very close to tofacitinib in spatial position. Thus, *α*‐MG may interfere with tofacitinib binding to CYP3A4 through spatial site‐blocking effects, which may be the mechanism of inhibition.

The above results provide some reference value for the combination of the two drugs and provide significant guidance for the clinical application of tofacitinib and *α*‐MG. Insufficiently, this study only provided the pharmacokinetic characteristics of tofacitinib under *α*‐MG coexistence from the rat model, which necessitates more clinical studies to verify the above findings.

In summary, this study confirmed HDI with *α*‐MG and tofacitinib. We also found that oral *α*‐MG enhanced tofacitinib exposure by inhibiting drug metabolism. Therefore, our findings suggest tofacitinib treatment for RA must include food and herbs containing *α*‐MG and the dose must be adjusted accordingly.

## Disclosure

The authors have nothing to report.

## Ethics Statement

The animal study was reviewed and approved by the Animal Ethics Committee of Wenzhou Medical University.

## Conflicts of Interest

The authors declare no conflicts of interest.

## Data Availability

The raw data supporting the conclusions of this article will be made available by the authors without undue reservation.
